# Total gastrectomy with simultaneous pancreaticosplenectomy or splenectomy in patients with advanced gastric carcinoma

**DOI:** 10.1038/sj.bjc.6690285

**Published:** 1999-04

**Authors:** E Otsuji, T Yamaguchi, K Sawai, K Okamoto, T Takahashi

**Affiliations:** First Department of Surgery, Kyoto Prefectural University of Medicine, Kawaramachi Hirokoji Kamigyo-ku, Kyoto, 602-0841, Japan

**Keywords:** gastric carcinoma, total gastrectomy, distal pancreatectomy

## Abstract

A splenectomy or distal pancreaticosplenectomy is often performed simultaneously with total gastrectomy in the treatment of gastric carcinoma to facilitate dissection of the lymph nodes around the splenic artery and splenic hilus. However, the negative impact of splenectomy and pancreaticosplenectomy has also been reported. A retrospective analysis was performed to evaluate the outcomes of distal pancreaticosplenectomy and total gastrectomy, splenectomy and total gastrectomy, and gastrectomy alone in the patients with advanced gastric carcinoma without distant metastasis. Prognostic factors were examined. No significant differences existed in 5-year survival in the patients who underwent gastrectomy with splenectomy, gastrectomy with distal pancreaticosplenectomy, or gastrectomy alone. Neither splenectomy, nor distal pancreaticosplenectomy were prognostic factors. However, distal pancreaticosplenectomy was an independent predictor of pancreatic fistula. In conclusion, the addition of distal pancreaticosplenectomy or splenectomy to total gastrectomy for gastric cancer increases the risk of severe complications, but does not improve survival. © 1999 Cancer Research Campaign


*British Journal of Cancer* (1999) **79**(11/12), 1789–1793
© 1999 Cancer Research Campaign
Article no. bjoc.1998.0285

### Total gastrectomy with simultaneous pancreaticosplenectomy or splenectomy in patients with advanced gastric carcinoma

**E Otsuji, T Yamaguchi, K Sawai, K Okamoto and T Takahashi**

First Department of Surgery, Kyoto Prefectural University of Medicine, Kawaramachi Hirokoji Kamigyo-ku, Kyoto 602-0841, Japan

**Summary** A splenectomy or distal pancreaticosplenectomy is often performed simultaneously with total gastrectomy in the treatment of gastric carcinoma to facilitate dissection of the lymph nodes around the splenic artery and splenic hilus. However, the negative impact of splenectomy and pancreaticosplenectomy has also been reported. A retrospective analysis was performed to evaluate the outcomes of distal pancreaticosplenectomy and total gastrectomy, splenectomy and total gastrectomy, and gastrectomy alone in the patients with advanced gastric carcinoma without distant metastasis. Prognostic factors were examined. No significant differences existed in 5-year survival in the patients who underwent gastrectomy with splenectomy, gastrectomy with distal pancreaticosplenectomy, or gastrectomy alone. Neither splenectomy, nor distal pancreaticosplenectomy were prognostic factors. However, distal pancreaticosplenectomy was an independent predictor of pancreatic fistula. In conclusion, the addition of distal pancreaticosplenectomy or splenectomy to total gastrectomy for gastric cancer increases the risk of severe complications, but does not improve survival.

**Keywords:** gastric carcinoma; total gastrectomy; distal pancreatectomy

### INTRODUCTION

The mortality from gastric carcinoma has recently decreased (Akoh and Macintyre, 1992). One of the reasons for the improved outcome is increased detection of early gastric cancers due to vigorous endoscopic screening (Kennedy, 1993). However, more than half of the gastric carcinomas detected are advanced (Bonenkamp et al, 1993). In the treatment of advanced carcinoma of the stomach, aggressive lymph node dissection in conjunction with gastrectomy has been reported to result in a substantial improvement in survival (Maruyama et al, 1987; Shiu et al, 1987). Splenectomy or distal pancreaticosplenectomy are often performed simultaneously with total gastrectomy to facilitate dissection of the lymph nodes around the splenic hilus and splenic artery. There have been reports of improved survival with added splenectomy or distal pancreaticosplenectomy compared with total gastrectomy alone in patients with gastric cancer (Noguchi et al, 1989; Takagi et al, 1980). However, the possibility of a negative impact of splenectomy or distal pancreatectomy has been raised (Sugimachiet et al, 1980; Yoshino et al, 1979; Suehiro et al, 1984; Maruyama, 1979; Otsuji et al, 1996). Because the spleen makes up 25% of the total lymphoid mass and is an important component of the reticuloendothelial system, there is an increased long-term risk of infection following splenectomy (Ellison et al, 1983). Although some Japanese surgeons have demonstrated that splenic hilar nodes can be completely dissected without splenectomy (Uyama et al, 1996), this procedure is not widely used. Several investigators have demonstrated that patients undergoing pancreaticosplenectomy in conjunction with total gastrectomy are at risk for leaks from the stump of the pancreas (Cuschieri et al, 1996). This predisposes to subphrenic abscess formation, dehiscence of the visceral anastomoses, and erosion of the blood vessels in the area of the pancreas, resulting in high peri-operative mortality rates. Thus, there is no consensus of opinion regarding the therapeutic value of splenectomy or distal pancreaticosplenectomy.

Clinically, it is thus difficult to decide whether simultaneous splenectomy or distal pancreaticosplenectomy should be performed with total gastrectomy in patients with advanced gastric carcinoma without distant metastasis. What is known, however, is that these patients have a high risk of lymph node metastasis around the splenic artery and splenic hilus.

To evaluate the effects of distal pancreaticosplenectomy and splenectomy on survival, a retrospective analysis of 128 patients who underwent total gastrectomy for gastric carcinoma was performed. The post-operative morbidity was compared between the patients who underwent distal pancreaticosplenectomy or splenectomy, and those who underwent gastrectomy alone.

*Received 28 May 1998*
*Revised 1 September 1998*
*Accepted 7 October 1998*

*Correspondence to:* E Otsuji

### PATIENTS AND METHODS

### Patients

Between 1983 and 1994, 128 patients underwent total gastrectomy for gastric carcinoma at the First Department of Surgery, Kyoto Prefectural University of Medicine, Kyoto, Japan. Of these patients, 46 (35.9%) underwent pancreaticosplenectomy and total

**Table 1** Clinicopathologic findings in the patients who underwent splenectomy and total gastrectomy with or without distal pancreatectomy

| Variables | Only total gastrectomy (*n* = 25) | With splenectomy (*n* = 57) | With pancreaticosplenectomy (*n* = 46) | *P* value |
|---|---|---|---|---|
| Age (year, mean) | 66.2 | 58.8 | 56.8 | NS |
| Sex (male/female) | 18/7 | 31/26 | 32/14 | NS |
| Tumour size (mm, mean) | 54.9 | 65.9 | 62.6 | NS |
| Primary tumour (pT2/pT3/pT4) | 13/9/3 | 32/22/3 | 23/19/4 | NS |
| Location (upper/middle/lower/whole) | 13/8/1/3 | 30/17/2/8 | 24/18/1/3 | NS |
| Circumference (greater/lesser/anterior/posterior/combined) | 9/4/2/4/6 | 24/8/9/8/8 | 21/5/8/6/6 | NS |
| Regional lymph nodes (positive/negative) | 17/8 | 35/22 | 32/14 | NS |
| UICC staging (Ia/Ib/II/IIIa/IIIb/IV) | 4/5/15/1 | 9/15/30/3 | 1/11/33/1 | NS |
| Residual tumour (R0/R1/R2) | 22/1/2 | 52/0/5 | 45/1/0 | NS |
| Operative time (min, mean) | 246 | 289 | 313 | < 0.05* |
| Pre-operative co-morbidity (positive/negative) | 15/10 | 32/25 | 29/17 | NS |
| Post-operative co-morbidity (positive/negative) | 9/16 | 23/34 | 21/25 | NS |
| Blood transfusion (positive/negative) | 14/11 | 33/24 | 27/19 | NS |

NS, not significant; *significant difference; greater, greater curvature; lesser, lesser curvature; anterior, anterior wall; posterior, posterior wall.

gastrectomy, 57 (44.6%) underwent splenectomy and total gastrectomy, and 25 (19.5%) underwent total gastrectomy alone.

### Surgical technique

The surgical procedures were performed by several attending surgeons on the faculty, or by the surgical fellows. Patients with gastric carcinoma located in the middle or proximal stomach underwent total gastrectomy by essentially the same technique. The definitions for classification were taken from the International Union Against Cancer (UICC) (Hermanek et al, 1987).

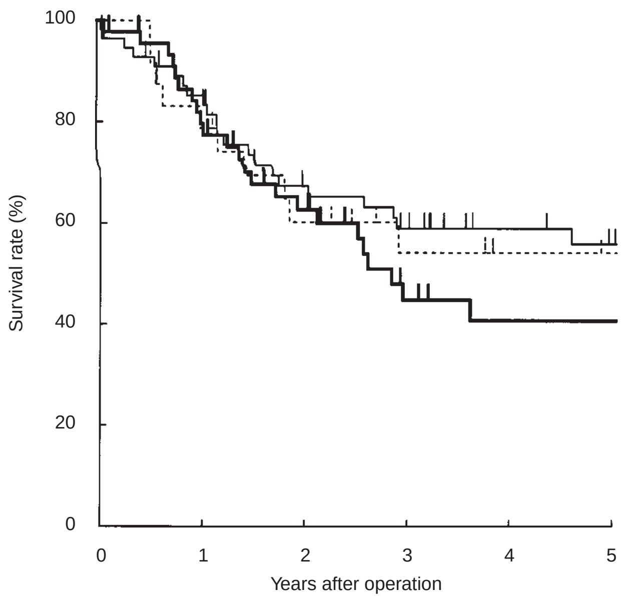

**Figure 1.** The 5-year survival rates for the patients who underwent total gastrectomy with or without splenectomy or pancreaticosplenectomy for gastric cancer. No significant differences existed between the survival rates in the three groups. ━ ━ ━, pancreaticosplenectomy with total gastrectomy; ——, splenectomy with total gastrectomy; - - -, total gastrectomy alone

### Clinicopathologic findings

Information collected from the medical records included the age and sex of the patient, as well as the size and location of the primary tumour, the depth of invasion, and whether regional lymph node metastases were present. The UICC stage, presence or absence of residual tumour, the operative time, pre-operative co-morbid conditions, and blood transfusion requirements were also recorded.

Post-operative complications were reviewed. A complication was not considered infectious unless confirmed by bacteriologic cultures.

### Statistical methods

Statistical analysis was performed using the NAP system (Version 4.0) programmed by Aoki (1989). The first objective of the statistical analysis was to examine the influence of each clinical, pathologic and treatment variable on survival following total gastrectomy. Information obtained from the univariate analysis (log-rank test) was applied to survival analysis with covariates

**Table 2** Univariate analysis of prognostic variables for survival

| Variables | *P*-value |
|---|---|
| Age (over or under 65 years old) | 0.21328 |
| Sex (female/male) | 0.18272 |
| Tumour size (over or under 5 cm) | 0.00180* |
| Primary tumour (pT1/pT2/pT3/pT4) | 0.00001* |
| Location (upper/middle/lower/whole) | 0.02212* |
| Regional lymph nodes (N0/N1/N2) | 0.00402* |
| Residual tumour (R0/R1/R2) | 0.00213* |
| Gross appearance (I/II/III/IV/other) | 0.01358* |
| Operative time (over or under 300 min) | 0.43562 |
| Pre-operative co-morbidity (positive/negative) | 0.87809 |
| Blood transfusion (positive/negative) | 0.18302 |

*Significant difference.

 © Cancer Research Campaign 1999

**Table 3** Multivariate analysis of prognostic variables for survival

| Variables | Regression coefficient | *P*-value |
|---|---|---|
| Depth of cancer invasion | 0.44759 | 0.00082* |
| Gross appearance | 0.42839 | 0.00279* |
| Residual tumour | 0.35851 | 0.00479* |
| Tumour size | 0.25439 | 0.15434 |
| Lymph node metastasis | 0.21016 | 0.15982 |
| Splenectomy or pancreaticosplenectomy | 0.08266 | 0.54194 |
| Location | 0.00421 | 0.97279 |

*Significant difference.

**Table 4** Post-operative complications following total gastrectomy

| Complication | Only total gastrectomy | With splenectomy | With pancreaticosplenectomy | *P*-value |
|---|---|---|---|---|
| Cardiac | 0 (0%) | 0 (0%) | 2 (4.3) | NS |
| Pulmonary | 1 (4.0%) | 2 (3.5%) | 0 (0) | NS |
| Liver dysfunction | 2 (8.0%) | 3 (5.3%) | 4 (8.7) | NS |
| Renal dysfunction | 0 (0%) | 1 (1.8%) | 0 (0) | NS |
| Bleeding | 1 (4%) | 0 (0%) | 1 (2.2) | NS |
| Anastomotic leakage | 3 (12.0%) | 8 (14.1%) | 7 (15.2) | NS |
| Intestinal obstruction | 1 (4.0%) | 2 (3.5%) | 0 (0) | NS |
| Pancreatitis | 0 (0%) | 1 (1.8%) | 1 (2.2) | NS |
| Pancreatic fistula | 1 (4.0%) | 1 (1.8%) | 7 (15.2) | 0.0271025* |
| Wound infection | 2 (8.0%) | 2 (3.5%) | 1 (2.2) | NS |

NS, not significant; *significant difference.

using the Cox model of proportional hazards (Cox, 1972). To analyse the influence of splenectomy or distal pancreatectomy on survival following gastrectomy, the Kaplan–Meier method and generalized Wilcoxon test were performed.

The second objective of the statistical analysis was to assess the incidence of the complications. The incidence of the post-operative co-morbidities was compared between groups using 11 variables.

### RESULTS

### Clinicopathologic findings

Significant differences were noted in the operative time between the groups (Table 1).

### Survival rates

The cumulative 5-year survivals of the patients who underwent total gastrectomy with distal pancreaticosplenectomy, total gastrectomy with splenectomy and total gastrectomy alone were 40.7%, 55.9% and 54.2% respectively. Using the generalized Wilcoxon test, a significant difference was not observed between the groups (Figure 1).

### Prognostic factors

Of the 13 clinical and pathologic variables identified by univariate analysis, six were found to be independent predictors of survival, and were selected for final proportional-hazards regression. Those variables were tumour size, depth of cancer invasion, location of the primary tumour, regional lymph node metastases, residual tumour, and the gross appearance of the tumour (Table 2). Multivariate analysis showed that significant prognostic factors were residual tumour, depth of cancer invasion and the gross appearance of the tumour. Neither distal pancreaticosplenectomy nor splenectomy were independent prognostic factors (Table 3).

### Complication following total gastrectomy

Pancreatic fistulae were significantly more common following total gastrectomy with distal pancreaticosplenectomy than after gastrectomy with splenectomy or total gastrectomy alone (Table 4).

### DISCUSSION

The extent of lymph node dissection with gastrectomy for gastric carcinoma has been a topic of much discussion (Gall et al, 1985;

© Cancer Research Campaign 1999

Soga et al, 1979, 1988). Lymphatic flow around the upper portion of the greater curvature of the stomach has been reported, toward the nodes around the splenic hilus and splenic artery (Takahashi et al, 1991). Yoshino et al have reported that spread of carcinoma is often to the lymph nodes around the splenic hilus and splenic artery when the primary tumour is located along the greater curvature of the upper third of the stomach (Yoshino et al, 1979). Simultaneous splenectomy and distal pancreaticosplenectomy have been advocated as standard procedures for proximal gastric cancer to facilitate removal of the lymph nodes around the splenic hilus and artery. The incidence of positive hilar node metastasis in patients with gastric carcinomas located in the upper third of the stomach has been reported to be greater than 25% (Fass and Schumpelick, 1989). In cases of curative total gastrectomy with splenectomy, the rate of cancerous involvement of the splenic hilar nodes was found to be about 10% (Sugimachi et al, 1980). In our study, the incidence of hilar nodal metastasis in patients who underwent splenectomy simultaneously with total gastrectomy for gastric carcinoma was 14% (14/103) (unpublished data).

Although splenectomy has been advocated for clearance of the splenic hilar lymph nodes, the importance of the spleen as a part of the immune system has only recently been stressed. The spleen is an important component of the reticuloendothelial system (Ellison and Fabri, 1983), and serves as a site of T- and B-lymphocyte interaction, which is important for the secondary immune response to foreign antigen challenges (Llende et al, 1986). Moreover, Griffith et al have reported that most of their patients with splenic hilar nodal metastasis had a primary tumour that had penetrated through the serosa, and had perigastric lymph node metastasis (Griffith et al, 1995). The prognosis in these patients was poor even after radical gastrectomy. Thus, whether or not to preserve the spleen has been vigorously debated.

In our previous study, the incidence of microscopic lymph node metastasis around the splenic artery in patients who underwent distal pancreatectomy simultaneously with total gastrectomy for gastric carcinoma was 15% (7/46) (unpublished data). Kanai (1967) has demonstrated, by examining sequential sections of the distal pancreas and surrounding tissues, that remnant nodes exist along the splenic artery in 74.7% of patients. This suggests that organ resection in the absence of true invasion is necessary to ensure the completeness of nodal dissection. However, Sugimachi et al (1982) have reported that many patients with nodal metastases at the splenic hilus or around the splenic artery are incurable with surgery because of factors other than the existence of these nodal metastases. Moreover, the morbidity after distal pancreaticosplenectomy and gastrectomy has been reported to be greater than that after splenectomy and gastrectomy, and gastrectomy alone (Fortner et al, 1994).

In the present study, we analysed patients with advanced gastric carcinoma without distant metastasis. It is often difficult to determine whether combined resection of the spleen or distal pancreas should be performed as a part of extended radical lymph node dissection. In this study, the 5-year survival rates of the patients who underwent distal pancreaticosplenectomy with total gastrectomy, splenectomy with total gastrectomy, or total gastrectomy alone were not statistically different. Moreover, neither pancreaticosplenectomy nor splenectomy were significant prognostic factors in the patients undergoing total gastrectomy.

The morbidity in patients who have undergone distal pancreaticosplenectomy with total gastrectomy has been reported to be greater than that after splenectomy and gastrectomy, or gastrectomy alone (Fortner et al, 1994; Otsuji et al, 1997). In this study, the incidence of pancreatic fistulae after distal pancreaticosplenectomy and total gastrectomy was significantly higher than that after splenectomy and total gastrectomy and total gastrectomy alone.

Because only the in-hospital morbidity was analysed in this study, the long-term risk of infection following splenectomy was not considered. Therefore, in patients with gastric cancer, the addition of distal pancreaticosplenectomy or splenectomy to total gastrectomy increases the risk of severe complication, but does not improve survival.

### REFERENCES

Akoh JA and Macintyre IMC (1992) Improving survival in gastric cancer: review of 5-year survival rates in English language publications from 1970. *Br J Surg* **79**: 193–199

Aoki S (1989) *Reference manual for the Medical Statistical Analysis*, 1st ed. Igakushoin: Tokyo

Bonenkamp JJ, Veld CJH, Kampschper GHM, Herman J, Hermanek P, Bemelmans M, Gouma DJ, Sasako M and Maruyama K (1993) Comparison of factors influencing the prognosis of Japanese, German, and Dutch gastric cancer patients. *World J Surg* **17**: 410–415

Cox DR (1972) Regression models and life tables. *J R Stat Assoc* **29**: 187–220

Cuschieri A, Fayers P, Fielding J, Craven J, Bancewicz J, Joypaul V and Cook P (1996) Postoperative morbidity and mortality after D1 and D2 resections for gastric cancer: preliminary results of the MRC randomized controlled surgical trial. *Lancet* **347**: 995–999

Ellison EC and Fabri PJ (1983) Complications of splenectomy. *Surg Clin North Am* **63**: 1313–1330

Fass J and Schumpelick V (1989) Principles of radical surgery in gastric carcinoma. *Hepatogastroenterol* **36**: 13–21

Fortner JG, Lauwers GY, Thaler HT, Concepcion R, Friendlander-Klar H, Kher U and Maclean BJ (1994) Nativity, complications, and pathology are determinants of surgical results for gastric cancer. *Cancer* **73**: 8–14

Gall FP and Hermanek P (1985) New aspects in the surgical treatment of gastric carcinoma. A comparable study of 1636 patients operated on between 1969 and 1982. *Eur J Surg Oncol* **11**: 219–225

Griffith JP, Sue-Ling HM, Martin I, Dixon MF, McMahon MJ, Axon ATR and Johnston D (1995) Preservation of the spleen improves survival after radical surgery for gastric cancer. *Gut* **36**: 684–690

Hermanek P and Sobin LH (1987) *TNM-classification of Malignant Tumors*, 4th ed. Springer: Berlin

Kanai H (1967) Significance of combined pancreaticosplenectomy in gastric resection for gastric carcinoma. *J Jpn Soc Cancer Ther* **2**: 328–338

Kennedy BJ (1993) Cure for early gastric cancer. *Cancer* **72**: 3139–3140

Llende M, Santiago-Delpin EA and Lavergne J (1986) Immunobiological consequences of splenectomy; a review. *J Surg Res* **40**: 85–94

Maruyama K (1979) A new dissection technique of superior pancreatic lymph nodes. *Jpn J Gastroenterol Surg* **12**: 961–965

Maruyama K, Okabayashi K and Kinoshita T (1987) Progress in gastric surgery in Japan and its limits of radicality. *World J Surg* **11**: 418–425

Noguchi Y, Imada T, Matsumoto A, Coit DG and Brennan MF (1989) Radical surgery for gastric cancer: a review of the Japanese experience. *Cancer* **64**: 2053–2062

Otsuji E, Yamaguchi T, Sawai K, Ohara M and Takahashi T (1996) End-results of simultaneous splenectomy in patients undergoing total gastrectomy for gastric carcinoma. *Surgery* **120**: 40–44

Otsuji E, Yamaguchi T, Sawai K, Okamoto K and Takahashi T (1997) End-results of simultaneous pancreatectomy, splenectomy and total gastrectomy for patients with gastric carcinoma. *Br J Cancer* **75**: 1219–1223

Shiu MH, Moore E, Sanders M, Huvos A, Freedman B, Goodbold J, Chaiyaphruk S, Wesdorp R and Brennan MF (1987) Influence of the extent of resection on survival after curative treatment of gastric carcinoma. *Arch Surg* **122**: 1347–1351

Soga J, Kobayashi K, Saito J, Fujimaki T and Muto T (1979) The role of lymphadenectomy in curative surgery for gastric cancer. *World J Surg* **3**: 701

Soga J, Ohyama S, Miyashiba, Suzuki T, Nashimoto A and Tanaka O (1988) A statistical evaluation of advancement of gastric cancer surgery with special reference to the significance of lymphadenectomy for cure. *World J Surg* **12**: 398–405

 © Cancer Research Campaign 1999

Suehiro S, Nagasue N, Ogawa Y, Sasaki Y, Hirose S and Yukawa H (1984) The negative effect of splenectomy on the prognosis of gastric cancer. *Am J Surg* **148**: 645–648

Sugimachi K, Kodama Y, Kumashiro R, Kanematsu T, Noda S and Inokuchi K (1980) Critical evaluation of prophylactic splenectomy in total gastrectomy for the stomach cancer. *Gann* **71**: 704–709

Sugimachi K, Kodama Y, Okamura K, Shiraishi M, Kuwano H and Inokuchi K (1982) Splenectomy in total gastrectomy: viewing against prophylactic splenectomy. *Operation* **36**: 337–343

Takahashi T, Sawai K, Hagiwara A, Takahashi S, Seiki K and Tokuda H (1991) Type-originated therapy for the gastric cancer effective for lymph node metastasis: management of lymph node metastasis using activated carbon particles adsorbing an anticancer agent. *Seminar Surg Oncol* **7**: 378–383

Takagi K, Ohashi I and Ohta K (1980) Significance of combined resection of adjacent organs for carcinoma of the stomach. *Surg Therapy* **12**: 667–675

Uyama I, Ogiwara H, Takahara T, Kikuchi K, Iida S, Kubota T, Kumai K and Kitajima M (1996) Spleen- and pancreas-preserving total gastrectomy with superextended lymphadenectomy including dissection of para-aortic lymph nodes for gastric cancer. *J Surg Oncol* **63**: 268–270

Yoshino K, Haruyama K, Nakamura S, Matsumoto S, Yamada Y, Isobe K, Kubota T, Kumai K, Ishibiki H and Abe O (1979) Evaluation of splenectomy for gastric carcinoma. *Jpn J Gastroenterol Surg* **12**: 944–949

*© Cancer Research Campaign 1999*
